# Characteristics and Specialist Linkage to Care of Patients Diagnosed With Chronic Hepatitis C Across Different Settings in an Urban Academic Hospital: Implications for Improving Diagnosis and Linkage to Care

**DOI:** 10.3389/fmicb.2021.576357

**Published:** 2021-02-11

**Authors:** Dan C. S. Im, Susheel Reddy, Claudia Hawkins, Shannon Galvin

**Affiliations:** Division of Infectious Diseases, Feinberg School of Medicine, Northwestern University, Chicago, IL, United States

**Keywords:** chronic hepatitis C, linkage to care, screening, hepatitis, hepatitis (C) virus

## Abstract

**Introduction:**

Chronic hepatitis C virus (HCV) infection is a significant public health problem. Strategies to identify more HCV infections and improve linkage to care (LTC) are needed. We compared characteristics, treatment and LTC among chronic HCV patients in different health care settings.

**Methods:**

Newly diagnosed HCV antibody positive (anti-HCV+) patients within settings of acute care, inpatient and outpatient in one health system were studied. Proportion of LTC and treatment were analyzed only for HCV RNA positive patients. Chi-square, one-way ANOVA and logistic regression were used to compare the characteristics and outcomes in the three care settings. Patients in acute care settings were excluded from multivariate analyses due to low sample size.

**Results:**

About 43, 368, and 1159 anti-HCV+ individuals were identified in acute care, inpatient, and outpatient, respectively. Proportion of RNA positivity in acute, inpatient, and outpatient were 47.8, 60.3 and 29.2%, respectively (*p* < 0.01). After adjusting for age, insurance type, race, and gender, outpatients had higher odds of LTC and of treatment (OR 4.7 [2.9, 7.6] and 4.5 [2.8, 7.3]).

**Conclusions:**

Inpatients had lower proportion of LTC and treatment compared to outpatients. Use of LTC coordinators and the provision of integrated service for specialty care may improve outcomes.

## Introduction

Chronic hepatitis C virus (HCV) is one of the main causes of chronic liver disease affecting approximately 130–170 million and 3 million individuals worldwide and in the United States, respectively (Lozano et al., 2012; Denniston et al., 2014). If left untreated, HCV infection can result in chronic infection in about 50–80% of individuals which may lead to end stage liver disease, hepatocellular carcinoma and other liver-related complications (Hoshida et al., 2014; Westbrook and Dusheiko, 2014). Untreated HCV can also pose a significant public health concern by increasing the risk of transmission (Urbanus et al., 2009).

Management of chronic HCV infection has advanced recently through a deeper understanding of the pathophysiology and introduction of direct acting oral antivirals (DAA). DAA has helped patients achieve sustained virologic response (SVR) which substantially reduced the liver-related complications such as fibrosis and cirrhosis (Ponziani et al., 2017). Despite the availability of effective treatments, a low number of HCV-infected individuals are aware of their infection status in United States. It has been reported that approximately 50% of HCV-infected persons are unaware of their infection. Prior to introduction of DAA, only an estimated 13–18% of HCV-infected persons had received treatment by 2013 (Denniston et al., 2012, 2014). A major barrier to this low proportion of treatment is access issues related to awareness, testing, specialist visit and medication cost. Attendance to specialist care after a positive HCV test and continued follow up can be used to understand patient’s determination to seek HCV treatment. Though the treatment regimen is different from the one of HCV, failure to show up to appointments has been linked to higher mortality due to medication non-adherence in HIV patients (Horberg et al., 2013). In two recent studies of HCV screening programs conducted in the emergency room, appointment attendance to specialty care after a positive HCV test was as low as 24%, and only a third of patients were successfully linked to HCV care (Franco et al., 2016; White et al., 2016). In another large study examining appointment keeping behaviors in HIV/HCV and HCV infected individuals in a large urban network of primary care clinics, 67% of patients kept at least one follow up appointment (Pundhir et al., 2016). Many reasons for such a result were identified including: perceived unimportance of diagnosis due to long referral time, lack of discussion by primary care physician, limited knowledge about HCV, feeling stigmatized, and lack of adequate financial resources (Zacks et al., 2006; Evon et al., 2010; Pundhir et al., 2016). Even in the era of DAA, there seem to be barriers between LTC and achieving SVR. In a study that followed patients in a single infectious disease clinic from referral to SVR, only 53% of the patients achieved SVR (Zuckerman et al., 2018). Addressing these issues therefore seems to be an important prerequisite to reduce HCV morbidity and mortality.

Although many strategies have been studied to effectively engage HCV-affected persons in acute care and outpatient settings, few have been examined in the inpatient setting. In non-clinical and clinical centers in the community, the use of patient navigators or linkage to care (LTC) coordinators were shown to be effective to diagnose, link, retain and re-engage patients in HCV care (Coyle et al., 2015; Trooskin et al., 2015). Furthermore, clinics or hospitals where patients can receive integrated services for HCV facilitated LTC by reducing the number of referrals and travel required to see different specialists (Ramirez et al., 2016). In the United States, HCV infection accounts for 475,000 inpatient hospital stays annually with baby boomer cohort (those born between 1945 and 1965) accounting for 70.7% of these admissions. Between 2001 and 2010, the number of inpatient stays increased by more than 60% which resulted in a significant economic burden, totaling approximately $6 billion annually (Galbraith et al., 2014). Inpatient admissions are therefore optimal settings to identify and link a large number of HCV-infected patients to care and re-engage any patients previously lost to follow up when compared to prior strategies focusing on persons already engaged in outpatient care.

With the availability of an effective cure that can lower the risk of complications and public health concerns strategies to identify and engage a larger number of undiagnosed HCV-infected individuals are needed urgently. While data exists on the HCV linkage outcomes in emergency department patients (Galbraith et al., 2015; Anderson et al., 2017), outpatients (Coyle et al., 2015) and inpatients (Turner et al., 2015; Taylor et al., 2016), very limited data exists on all three settings from one cohort (Calner et al., 2019) and even less information about what characteristics from patients in different settings within one system are associated with LTC. Here we seek to characterize and differentiate the demographics, risk factors and outcomes of persons identified with HCV across three different medical settings at one institution: inpatient, outpatient and an acute care setting such as the emergency department. Proportion of LTC as well as treatment were compared in those care settings as well. This information can be used to design screening strategies that are most effective and address patient needs across differing medical settings and patient populations, including inpatients.

## Materials and Methods

### Study Design

We conducted a retrospective cross-sectional study in the Northwestern Memorial Hospital (NMH) network system. NMH is a large, urban and tertiary university hospital located in Chicago, IL, United States. The study population was unique adults (18 or older) with positive hepatitis C antibody (anti-HCV+) result from tests conducted during the past three years (2/17/2015 – 2/17/2018) in the NMH network. Subjects were further categorized into three groups: inpatient, outpatient and acute care (emergency room, immediate care or observation unit) based on the location of the HCV antibody test order. Northwestern’s Enterprise Data Warehouse (EDW) provided the following information for each patient: age; gender; self-reported race; insurance status and type; last available HIV antibody; last available HBV surface antigen; last available HBV core antibody; last available HBV surface antibody; date of hepatitis B diagnosis; scheduled appointment at hepatology or infectious diseases and date of follow up appointment; receipt of any currently available DAA; HCV genotype; HCV RNA; platelet count; ALT; and AST. For all patients identified by EDW, medical record numbers were requested to review their charts for intravenous (IV) drug use (IVDU) history. The primary outcome was proportion of LTC among HCV RNA+ patients as defined by at least one scheduled appointment with hepatology or infectious diseases, secondary analysis included evaluation for differences between groups in the demographics (age, race, and gender); HCV risk factors (IVDU, birth cohort 1945–1965); proportion of HCV antibody positive with available RNA; initiation of hepatitis C-specific therapy as determined by prescription records. In the Northwestern medical system, specialists often initiate HCV specific care as opposed to primary physicians and thus linkage to specialty care was seen as good indicator for linkage to treatment, however receipt of HCV therapy was assessed independently of linkage to specialty care. The study was reviewed and approved by the Northwestern University Institutional Review Board.

### Statistical Analysis

Descriptive statistics was used to describe overall demographics. One-way ANOVA was used to compare continuous data between groups, chi-square methodologies was utilized to assess categorical data. For multivariate analysis, logistic regression was used to assess adjusted associations between care settings, gender, race (black, white, or other), insurance type (public, private or self-pay) and age per 10 years while controlling for one another. With this, it was attempted to adjust the outcome for important factors contributing to social determinants of health.

## Results

### Demographics

A total of 1,570 patients were identified as anti-HCV+. Among those, 43, 368, and 1,159 patients were identified in acute, inpatient and outpatient settings respectively. The demographics of these population can be found in Table 1. The patient population in acute, inpatient and outpatients were heterogeneous with significantly different proportions in gender, race, and age (Table 1). The proportion of patients belonging in the birth cohort 1945–1965 was 39.5, 60.6, and 64.8% (*p* < 0.01) in acute, inpatient, and outpatient settings, respectively. The most common type of insurance in acute (61.9%) and inpatients (75.1%) were public insurances such as Medicare and Medicaid while in outpatients, it was private (58.3%, *p* < 0.01).

**TABLE 1:** Demographics of the patient sample population.

	Acute	Inpatient	Outpatient	Total	*P* value
	(*N* = 43)	(*N* = 368)	(*N* = 1159)	(*N* = 1570)	
Ethnicity – *n* (%)	42 (97.7)	332 (90.2)	1015 (87.6)	1389 (88.5)	0.51
Hispanic	4 (9.5)	40 (12.0)	100 (9.9)	144 (10.4)	
Not Hispanic	38 (90.5)	292 (88.0)	915 (90.1)	1245 (89.6)	
Gender – *n* (%)	43 (100)	368 (100)	1159 (100)	1389 (100)	<0.01
Male	21 (48.8)	241 (65.5)	653 (56.3)	915 (58.3)	
Female	22 (51.2)	127 (34.5)	506 (43.7)	655 (41.7)	
Race – *n* (%)	42 (97.7)	354 (96.2)	1073 (92.6)	1469 (93.6)	<0.01
Asian	1 (2.4)	5 (1.5)	40 (3.9)	46 (3.3)	
Black	12 (28.6)	117 (35.5)	263 (25.4)	392 (27.9)	
White	24 (57.1)	173 (52.4)	650 (62.8)	847 (60.2)	
Other	5 (11.9)	35 (10.6)	82 (7.9)	122 (8.7)	
Birth Cohort 1945-65 – *n* (%)	43 (100)	368 (100)	1159 (100)	1570 (100)	<0.01
Yes	17 (39.5)	223 (60.6)	751 (64.8)	991 (63.1)	
No	26 (60.5)	145 (39.4)	408 (35.2)	579 (36.9)	
Insurance – *n* (%)	42 (97.7)	354 (96.2)	1073 (92.6)	1469 (93.6)	<0.01
Private	13 (31.0)	70 (19.8)	625 (58.3)	708 (48.2)	
Public	26 (61.9)	266 (75.1)	397 (37.0)	689 (46.9)	
Self-pay	3 (7.1)	18 (5.1)	51 (4.8)	72 (0.1)	
Age (years)					<0.01
Mean (SD)	46.0 (15.08)	55.7 (14.39)	54.5 (13.74)		

### Characteristics of HCV RNA Positive Patients

Among the Anti-HCV+ patients, 53.5% of acute, 79.6% of inpatients, and 88.9% of outpatients had an order for HCV RNA (*p* < 0.01, Table 2). Of those, 47.8% (*n* = 11) of acute, 60.3% (*n* = 176) of inpatients and 29.2% (*n* = 297) of outpatients had a positive RNA results (*p* < 0.01). Given low sample size of HCV RNA positive persons in acute care setting, only inpatients and outpatients were compared for further analysis. HCV RNA positive persons in inpatient and outpatient did not differ by age, HCV genotype or history of IV drug use (*p* = 0.11, 0.57, and 0.09, respectively). There was no difference in the proportion of HIV seropositivity between groups or markers of current and/or past co-infection with HBV.

**TABLE 2:** Characteristics of HCV RNA Positive patients.

	Acute	Inpatient	Outpatient	Total	*P* value
	(*N* = 43)	(*N* = 368)	(*N* = 1159)	(*N* = 1570)	
HCV RNA test ordered – *n* (%)	43 (100)	368 (100)	1159 (100)	1570 (100)	<0.0001
Yes	23 (53.5)	293 (79.6)	1030 (88.9)	1346 (85.7)	
No	20 (46.5)	75 (20.4)	129 (11.1)	224 (14.3)	
HCV RNA – *n* (%)	23 (53.5)	293 (79.6)	1030 (88.9)	1346 (85.7)	<0.0001
Positive	11 (47.8)	176 (60.3)	297 (29.2)	484 (36.0)	
Negative	12 (52.2)	117 (39.7)	733 (70.8)	862 (64.0)	
Log HCV RNA among HCV RNA positives					0.22
Mean (SD)	13.2 (2.27)	13.4 (2.43)	13.7 (2.2)		
Age among HCV RNA positives					0.11
Mean (SD)	43.8 (13.2)	55.8 (13.4)	57.8 (11.8)		
Genotypes – *n* (%)	3 (7.0)	74 (20.1)	206 (17.8)	283 (18.0)	0.57^a^
1	3 (100)	52 (70.3)	165 (80.1)	220 (77.7)	
2	0 (0)	10 (13.5)	17 (8.3)	27 (9.5)	
3	0 (0)	10 (13.5)	18 (8.7)	28 (9.9)	
4	0 (0)	2 (2.7)	4 (1.9)	6 (2.1)	
6	0 (0)	0 (0)	2 (1.0)	2 (0.7)	
Drug use among HCV RNA positives – *n* (%)	7 (63.6)	161 (91.5)	263 (88.6)	431 (89.1)	0.09^a^
IV	4 (57.1)	38 (23.6)	58 (22.1)	100 (23.2)	
Unknown Route	0 (0)	20 (12.4)	19 (7.2)	39 (9.1)	
Never Used	3 (42.9)	103 (64.0)	186 (70.7)	292 (67.8)	
HIV Ag/Ab among HCV RNA positives	5 (45.5)	110 (62.5)	175 (58.9)	290 (59.9)	0.44^a^
Reactive	0 (0)	2 (1.8)	8 (4.6)	10 (3.5)	
Non-reactive	5 (100)	108 (98.2)	167 (95.4)	280 (96.5)	
HBsAG among HCV RNA positives	11 (100)	156 (88.6)	241 (81.1)	408 (84.3)	0.18^a^
Reactive	1 (9.1)	1 (0.6)	5 (2.1)	7 (1.7)	
Non-reactive	10 (90.9)	155 (99.4)	236 (97.9)	401 (98.3)	
AntiHBs among HCV RNA positives	10 (90.9)	120 (68.2)	223 (75.1)	353 (72.9)	0.62^a^
Reactive	4 (40)	34 (28.3)	76 (34.1)	114 (32.3)	
Grayzone	1 (10)	8 (6.7)	9 (4.0)	18 (5.1)	
Non-reactive	5 (50)	78 (65.0)	138 (61.9)	221 (62.6)	
AntiHBc among HCV RNA positives	5 (45.5)	109 (61.9)	211 (94.6)	325 (67.2)	0.42^a^
Reactive	3 (60.0)	41 (37.6)	71 (33.7)	115 (35.4)	
Non-reactive	2 (40.0)	68 (62.4)	140 (66.3)	210 (64.6)	
ALT among HCV RNA positives					<0.0001
Mean (SD)	164.1 (220.9)	124.1 (363.3)	67.8 (153.5)		
AST among HCV RNA positives					<0.0001
Mean (SD)	114.7 (162.7)	153.2 (470)	62 (92)		
Platelet among HCV RNA positives					<0.0001
Mean (SD)	224.3 (100.8)	192.6 (154.5)	206 (85.6)		

*Ag, antigen; Ab, antibody; HBsAg, Hepatitis B surface antigen; AntiHBs, Hepatitis B surface antibody; AntiHBc, hepatitis B core antibody; ALT, Alanine aminotransferase (units/L); AST, Aspartate aminotransferase (units/L). ^*a*^Exact methodology applied.*

### Linkage to Care and Treatment

We then evaluated the LTC amongst HCV RNA+ individuals by searching for any scheduled, whether attended or not, appointment with Infectious Diseases (ID) or Hepatology following the positive HCV antibody test. Again, given the low sample size of HCV RNA positivity in acute care settings, the analysis focused only on inpatient vs. outpatient settings. LTC was higher in outpatients with 193/297 (65.0%) HCV RNA positive persons compared to 53/176 (30.1%) inpatients (*p* < 0.01). Proportion of treatment, as defined by proportion of HCV RNA+ persons on DAA during the study time period, was higher in outpatients with 187/297 (63.0%) individuals as opposed to 42/176 (23.9%) inpatients (*p* < 0.01, Table 3).

**TABLE 3:** Bivariate analysis of Linkage to Care (LTC) and medication use (proportion of treatment) among HCV RNA positive patients.

	Acute	Inpatient	Outpatient	Total	*P* value
	(*N* = 43)	(*N* = 368)	(*N* = 1159)	(*N* = 1570)	
Infectious Diseases or hepatology appointment among HCV RNA positives	11 (25.6)	176 (47.8)	297 (25.6)	484 (30.8)	<0.0001
Yes	3 (27.3)	53 (30.1)	193 (65.0)	249 (51.5)	
No	8 (72.7)	123 (69.9)	104 (35.0)	235 (48.5)	
HCV medication usage among HCV RNA positives	11 (25.6)	176 (47.8)	297 (25.6)	484 (30.8)	<0.0001
Yes	2 (18.2)	42 (23.9)	187 (63.0)	231 (47.8)	
No	9 (81.8)	134 (76.1)	110 (37.0)	253 (52.2)	

### Multivariate Analysis

The results of multivariate analysis are summarized in Table 4. Multivariate analysis of HCV RNA status amongst those who had positive hepatitis C antibody revealed that outpatients had reduced odds of a positive results when compared to inpatients (Odds ratio (OR) 0.37, [0.27, 0.50]). However, there was no difference when comparing acute care setting with inpatient (OR = 0.89, [0.36, 2.3]) or outpatients with acute (OR 0.41, [0.17, 1.0]). Other risk factors associated with higher odds of HCV RNA positivity included: male gender (OR 1.7, [1.3, 2.2]), Black race (OR 2.2, [1.7, 3.0] when compared to white race, OR 2.5, [1.6, 4.0] when compared to Other), Public insurance and Self-pay when compared to private insurance (OR 1.9, [1.5, 2.6] and OR 2.8, [1.5, 5.0], respectively).

**TABLE 4:** Multivariate analysis of HCV RNA positivity, proportion of linkage to care, and medication usage (treatment).

	HCV RNA positivity	Linkage to care	Medication usage
Acute: Inpatient	0.89 (0.36, 2.3)	–^†^	–^†^
Outpatient: Inpatient	*0.37 (0.27, 0.50)*	*4.9 (3.1, 7.9)*	*4.6 (2.9, 7.3)*
Outpatient: Acute	0.41 (0.17, 1.0)	–^†^	–^†^
Male: Female	*1.7 (1.3, 2.2)*	0.87 (0.55, 1.4)	*0.56 (0.35, 0.89)*
Black: White	*2.2 (1.7, 3.0)*	*2.0 (1.3, 3.3)*	1.0 (0.64, 1.6)
Black: Other	*2.5 (1.6, 4.0)*	1.0 (0.44, 2.3)	2.2 (0.94, 5.2)
Other: White	0.90 (0.57, 1.4)	2.0 (0.89, 4.5)	0.48 (0.20, 1.1)
Public: Private	*1.9 (1.5, 2.6)*	0.90 (0.54, 1.5)	*0.52 (0.31, 0.85)*
Self-pay: Private	*2.8 (1.5, 5.0)*	0.72 (0.28, 1.9)	0.44 (0.17, 1.2)
Public: Self-pay	0.71 (0.38, 1.3)	1.3 (0.47, 3.3)	1.2 (0.45, 3.0)
Age (per 10 years)	1.1 (0.97, 1.2)	1.1 (0.92, 1.3)	*1.2 (1.03, 1.5)*

*^†^Multivariate analysis not performed due to low sample size. Values in italics are statistically significant.*

Multivariate analysis of LTC amongst HCV RNA positive individuals showed that outpatients had higher odds of LTC compared to inpatients (OR 4.9, [3.1, 7.9]) when adjusted for race, insurance type and age per 10 years. The other association with higher LTC was black race (OR 2.0, [1.3, 3.3]). Similarly, outpatients were also more likely to receive DAA than inpatients (OR 4.6, [2.9, 7.3]). Increasing age was also associated with higher proportion of treatment (OR 1.2, [1.03, 1.5]). Male gender and having public insurance compared to private insurance were associated with lower proportion of treatment (OR 0.56, [0.35, 0.89] and OR 0.52, [0.31, 0.85], respectively).

## Discussion

In this study, we have shown that different care settings at the time of hepatitis C diagnosis were associated with signification variation in patient demographics, HCV RNA positivity, proportion of LTC and likelihood of receiving DAA. Most importantly, acute and inpatients were more likely to have positive RNA status while proportion of LTC and treatment was significantly lower in inpatients compared to outpatients. In the era of DAA, this provides potential opportunity for improving LTC and treatment access and thus cure. among patients in acute and inpatient settings.

There could be many reasons for lower proportion of LTC in inpatients. They may be less likely than outpatients to have a primary physician to help them navigate care. Inpatient patients tend to be discharged with more specialist appointments compared to patients seen in outpatient settings. This may burden patients and cause poor outcomes in LTC. Furthermore, they are often asked to follow up many non-urgent abnormal lab values such as positive hepatitis C antibody that are found during hospitalization as an outpatient. It is also possible that there needs to be a more rigorous care managing and response team to positive antibody test to prevent patients from falling off the care continuum. In addition, differences in health status and co-morbidities among inpatients as compared to outpatients may have affected initiation of HCV therapies and possibly even linkage to HCV care.

The need for more rigorous HCV screening and LTC in acute care and inpatient settings has been previously identified (Assoumou et al., 2014). Multiple studies in emergency department setting have shown low proportion of LTC and treatment (Allison et al., 2016; Franco et al., 2016). Even though our sample size for acute care setting was not large enough to allow us to investigate proportion of the LTC and treatment, our data suggests that patients in acute care setting are just as likely to have positive RNA status as inpatients but lower proportion of screening which re-emphasizes the need for better screening strategies in acute care. Few studies have examined outcomes of hospital-based HCV diagnosis, but in one study involving inpatients at a South Texas safety-net hospital, proportion of LTC was 44% and treatment was 7% (Taylor et al., 2016). Our study showed similarly low proportion of treatment and LTC.

The results of multivariate analysis shed some light into who is more likely to be linked to care and start medication. In general, male gender, black race, public insurance, and self-pay was more associated with positive RNA status indicating current infection. This is not surprising if one considers the socioeconomic status influencing access to healthcare and the fact that hepatitis C disproportionately affects African Americans (Miller et al., 2016). In fact, it has been previously reported that access to primary care, which can serve as a surrogate of healthcare access in general, is associated with higher proportion of successful LTC (Franco et al., 2016). This result is further complicated by the fact that male gender and public insurance holders were associated with lower proportions of treatment. This warrants more aggressive screening strategies and LTC coordination for male, African Americans, and people with public or no insurance. Interestingly however, black race was the only factor associated with higher LTC among patients with positive HCV RNA but similar proportion of treatment compared to other races. Our speculation is that due to the increased prevalence and concern of hepatitis C among this group, African American patients may be more likely to follow up with a specialist, but the cost of DAA may be a hindrance to the completion of care across groups.

Some strategies have been previously found to be effective in promoting LTC and treatment. Provision of HCV treatment by primary care providers can help improve access, but this still requires linkage or re-linkage to a primary care provider for those diagnosed in acute or inpatient settings. The utilization of electronic health record clinical decision support tool to remind physicians to screen baby boomers for HCV with care coordination has been effective in outpatient clinics (Castrejón et al., 2017). In inpatient settings, a program that included clinician education, electronic health record algorithm for eligibility and order entry, opt-out consent, personalized inpatient counseling and outpatient case management have been found to result in greater than 80% of LTC (Turner et al., 2015). Our institution has recently implemented a program for inpatients that facilitates rapid diagnosis and LTC through immediate evaluation by infectious diseases or hepatology physicians, patient education, assistance in appointment scheduling and follow up for missed appointments. Further research will be necessary to determine if this strategy will improve LTC for patients newly diagnosed in inpatient setting.

Limitations of this study include that this is a retrospective study based on electronic medical records data. While this study provides perspectives in the area that healthcare system needs to concentrate resources, there needs to be more work in determining the root cause of the poor outcome in certain population over others. It is difficult to categorize the socioeconomic status and social determinants of health that heavily influence the treatment with categorical data available in medical records. Additionally, differences in notification to patients of positive HCV antibody results between outpatient, inpatient and acute care settings could not be assessed and may have affected LTC outcomes. Sample size for those who tested positive for HCV RNA in acute care setting was also not enough for a meaningful comparison.

## Data Availability Statement

The raw data supporting the conclusions of this article will be made available by the authors, without undue reservation.

## Ethics Statement

The studies involving human participants were reviewed and approved by Northwestern University Institutional Review Board. Written informed consent for participation was not required for this study in accordance with the national legislation and the institutional requirements.

## Author Contributions

DI contributed in study design, data collection and analysis, and manuscript draft. SR contributed in data collection and analysis. CH and SG contributed in study conception, design, data analysis, and manuscript revision. All authors contributed to the article and approved the submitted version.

## Conflict of Interest

The authors declare that the research was conducted in the absence of any commercial or financial relationships that could be construed as a potential conflict of interest.
